# Ambulatory COVID-19 Patients Treated with Lactoferrin as a Supplementary Antiviral Agent: A Preliminary Study

**DOI:** 10.3390/jcm10184276

**Published:** 2021-09-21

**Authors:** Luigi Rosa, Giovanni Tripepi, Enrico Naldi, Marina Aimati, Stefano Santangeli, Francesco Venditto, Marcello Caldarelli, Piera Valenti

**Affiliations:** 1 Department of Public Health and Infectious Diseases, University of Rome “La Sapienza”, 00185 Rome, Italy; piera.valenti@uniroma1.it; 2 National Research Council (CNR), Institute of Clinical Physiology (IFC), Clinical Epidemiology of Renal Diseases and Hypertension, Ospedali Riuniti, 89124 Reggio Calabria, Italy; gtripepi@ifc.cnr.it; 3 General Practitioner, 50136 Florence, Italy; enrico.naldi60@virgilio.it; 4 General Practitioner, 04011 Aprilia, Italy; marina.aimati@gmail.com; 5 General Practitioner, Barberino del Mugello, 50031 Florence, Italy; dott.santangeli@alice.it; 6 General Practitioner, 50124 Florence, Italy; fravend@katamail.com; 7 General Practitioner, 00178 Rome, Italy; studio.drcaldm@gmail.com

**Keywords:** lactoferrin, COVID-19 patients, SARS-CoV-2

## Abstract

SARS-CoV-2, an enveloped, single-stranded RNA virus causing COVID-19, exerts morbidity and mortality especially in elderly, obese individuals and those suffering from chronic conditions. In addition to the availability of vaccines and the limited efficacy of the first dose of vaccine against SARS-CoV-2 variants, there is an urgent requirement for the discovery and development of supplementary antiviral agents. Lactoferrin (Lf), a pleiotropic cationic glycoprotein of innate immunity, has been proposed as a safe treatment combined with other therapies in COVID-19 patients. Here, we present a small retrospective study on asymptomatic, paucisymptomatic, and moderate symptomatic COVID-19 Lf-treated versus Lf-untreated patients. The time required to achieve SARS-CoV-2 RNA negativization in Lf-treated patients (*n* = 82) was significantly lower (*p* < 0.001) compared to that observed in Lf-untreated ones (*n* = 39) (15 versus 24 days). A link among reduction in symptoms, age, and Lf treatment was found. The Lf antiviral activity could be explained through the interaction with SARS-CoV-2 spike, the binding with heparan sulfate proteoglycans of cells, and the anti-inflammatory activity associated with the restoration of iron homeostasis disorders, which favor viral infection/replication. Lf could be an important supplementary treatment in counteracting SARS-CoV-2 infection, as it is also safe and well-tolerated by all treated patients.

## 1. Introduction

Severe Acute Respiratory Syndrome Coronavirus 2 (SARS-CoV-2), belonging to the family of Coronaviridae, is an enveloped, positive, single-stranded RNA virus that can infect animals and humans. As of 8 September 2021, there are 221,936,662 confirmed cases of SARS-CoV-2 infection in the world, with more than 4,586,100 deaths. The Istituto Superiore di Sanità reported that in Italy, there were 4,574,118 cases of confirmed SARS-CoV-2 infection, with more than 142,200 deaths.

The host receptor identified for SARS-CoV-2 is angiotensin-converting enzyme 2 (ACE2) [1,2]. Similar to another coronavirus as SARS-CoV, the receptor-binding domains of spike glycoproteins for SARS-CoV-2 recognize human ACE2 [3,4,5,6,7]. In humans, high ACE2 expression is found in alveolar epithelial cells, on the enterocytes of the ileum and colon, and on myocardial cells [8]. It is also present in vasal endothelial cells and in many other sites and organs such as the oral mucosa [9], nasal cavity, brain [10], and some hematopoietic cells, including monocytes and macrophages [11]. The localization of virus is closely related to the distribution of ACE2. Consequently, even if COVID-19 is recognized as a disease initially affecting the lungs, it is now clear that SARS-CoV-2 can also affect other organs, thereby increasing its severity [12,13,14].

Similar to other enveloped viruses [15] and to SARS-CoV [16], the cellular heparan sulfate proteoglycans (HSPGs), possessing a negative charge, interact electrostatically with basic residues of viral surface spike glycoproteins, strongly contributing to the early interaction between SARS-CoV-2 and host cells [17,18,19,20,21,22].

SARS-CoV-2 induces symptoms ranging from the common cold to severe respiratory distress syndrome, sepsis, and coagulopathy. The most common symptoms are fever, cough, dyspnea, headache, myalgia, and fatigue. Other reported symptoms are dysgeusia and anosmia [23]. Less common symptoms include diarrhea, hemoptysis, and shortness of breath [24]. Conversely, among more severe symptoms, coagulopathy has been reported in up to 50% of patients, as well as an increase in d-dimer levels, the most significant change in coagulation parameters observed in severe COVID-19 manifestations [25]. There is also evidence that SARS-CoV-2 can trigger the release of proinflammatory cytokines such as interleukin (IL)-6 [26,27], which, in turn, can also lead to coagulopathy [25,28]. Of note, high levels of proinflammatory cytokines and the consequent associated disorders are related to more severe events and disease progression [25,26,27,28].

Moreover, proinflammatory cytokines, particularly IL-6, can markedly influence iron homeostasis, which leads to intracellular iron overload [29]. In this respect, in the last year there has been a renewed interest in natural substances with antiviral and anti-inflammatory activity such as lactoferrin (Lf) [30,31], which could be an ideal candidate for simultaneously counteracting SARS-CoV-2 infection, inflammation, and iron homeostasis dysregulation [32,33,34,35].

Lf, an iron-binding glycoprotein belonging to the transferrin family, is formed by two lobes, lobe N and lobe C, each able to chelate one ferric ion [30]. Lf, synthesized by exocrine glands and neutrophils, is present in all human secretion, thus representing one of the most important glycoproteins of innate defenses of the host [30,36,37,38].

Lf guarantees that the quantity of free available iron does not exceed the concentration of 10^−18^ M to avoid microbial infection, replication, and the production of reactive oxygen species (ROS) [30,31,39].

This glycoprotein possesses an isoelectric point of about 9, responsible for its capacity to bind to anionic surface compounds of host cells, bacteria, and viruses, which hinders bacterial and viral entry into host cells [15,30,31].

Therefore, the ability of Lf to chelate two ferric ions per molecule as well as bind to anionic microbial and host cell surface structures could justify its antiviral activity.

Recently, it has been demonstrated that Lf is pivotal in modulating iron and inflammatory homeostasis [31], thus exerting a potent anti-inflammatory activity that rebalances iron homeostasis disorders, as demonstrated by a decrease in IL-6 synthesis in vitro [29] and in vivo [33,40,41].

For several years now, various products containing Lf have been commercialized as nutraceutical products. All of these products contain Lf extracted from bovine milk (bLf), which possesses a high homology of sequence with and similar functions to human Lf (hLf). Furthermore, bLf has been approved as a Generally Recognized as Safe (GRAS) compound by the United States Food and Drug Administration (USA FDA) [42] and as a dietary supplement by the European Food Safety Authority [43].

The potent antiviral action of bLf directed against many RNA, DNA, and enveloped or naked viruses is also well documented [15,44].

In the last year, there has been accumulating evidence from studies in vitro that bLf is active against SARS-CoV-2 [22,34,45]. BLf exerts its antiviral activity either by obscuring the host cell receptors [22] or by directly binding to the SARS-CoV-2 spike glycoproteins [34]. These in vitro results were confirmed by the in vivo study conducted by Campione et al. [33]. Thirty-two asymptomatic and mild-to-moderate COVID-19 patients, treated with liposomal bLf, showed a rapid recovery of clinical symptoms, an early reverse transcriptase real-time (rRT)-PCR SARS-CoV-2 RNA negative conversion, and a decrease in inflammatory markers such as IL-6, d-dimer, and serum ferritin compared to standard of care treatment [33].

Here, we present the results of a survey based on real-life clinical practice, conducted by Italian general practitioners on their asymptomatic, paucisymptomatic, and moderate symptomatic COVID-19 patients, in home-based isolation, treated with bLf unloaded in liposome, alone or as supplementary treatment, depending on subject’s symptoms. These results have been compared with those observed in COVID-19 patients untreated with bLf.

## 2. Materials and Methods

### 2.1. Setting

This was a survey based on real-life clinical practice in bLf-treated patients compared to a group of bLf-untreated ones. Data were retrospectively collected by some general practitioners located in the Tuscany and Lazio regions of Italy. For several years, bLf has been commercialized in Italy as a nutraceutical product. Ethical approval was not necessary according to National Code on Clinical Trials declaration [46], because our observation derives from a real-life retrospective study. All patients affected by SARS-CoV-2 infection were included in the study. Although allergy to milk proteins was specifically considered as the sole exclusion criterion in the study, no patient was excluded for this reason.

The objective of the present study was to observe the time of rRT-PCR SARS-CoV-2 RNA negative conversion in both groups of subjects (bLf-treated versus untreated). Data on the time to symptom remission and on hospitalization were also gathered. Moreover, safety and tolerability of bLf were also evaluated.

### 2.2. Study Population

From October 2020 to March 2021 all asymptomatic, paucisymptomatic, and moderate symptomatic confirmed COVID-19 patients with positive rRT-PCR nasopharyngeal swab were included in this retrospective survey. Patients ranged in age from 17 to 104 years. Data for each patient were collected.

Patients defined as “asymptomatic” were those patients who did not show any symptoms even if positive for COVID-19 infection. “Paucisymptomatics” were defined as subjects with low fever (<38 °C) and/or cough and/or sore throat and/or headache and/or general discomfort and/or myalgia. “Moderate symptomatics” included subjects with high fever (>38 °C for at least three days) and/or persistent cough and/or prolonged asthenia and/or widespread pains and/or dyspnea and/or gastro-intestinal problems and/or arthralgia and/or decreased blood oxygen saturation.

In all asymptomatic patients, general practitioners started bLf treatment after a positive rRT-PCR nasopharyngeal swab. In all symptomatic patients, this treatment started at the onset of first symptoms, considering the early treatment as the strategy to counteract any possible avoidable complications. Therefore, COVID-19 patients in home-based isolation were immediately treated with oral bLf alone or, when necessary, also received other medication (ibuprofen, paracetamol, azithromycin, heparin, and cortisone) depending on the conditions of the specific patient in order to optimize the home-based treatment.

The control group of COVID-19 patients were treated with the above-mentioned drugs without oral bLf.

All patients suffering from COVID-19 were monitored until complete remission of symptoms and until swab SARS-CoV-2 RNA negativization.

### 2.3. Patients’ Treatments

All COVID-19 patients were in home-based isolation.

In the bLf-untreated group, the paucisymptomatic and moderate symptomatic patients were treated with paracetamol 1000 mg 3 times/day until fever subsided and/or ibuprofen 600 mg 2 times/day until the remission of symptoms and/or cortisone 25 mg/day until the remission of symptoms and/or azithromycin 500 mg/day for 6 days, while asymptomatic patients received no treatment.

The bLf-treated group, including asymptomatic, paucisymptomatic, and moderate symptomatic patients, were treated with bLf from one to five capsules/day, each containing 200 mg of bLf (Mosiac^®^, Pharmaguida, Rome, Italy). The purity of bLf, checked by SDS-PAGE and silver nitrate staining, was 98%. The concentration of bLf was assessed by UV spectroscopy on the basis of an extinction coefficient of 15.1 (280 nm, 1% solution). The bLf iron saturation was about 7%, as detected by optical spectroscopy at 468 nm on the basis of an extinction coefficient of 0.54 (100% iron saturation, 1% solution).

The patients were treated with bLf until SARS-CoV-2 RNA negativization. Depending upon the absence/presence of symptoms related to COVID-19 infection and preexisting pathologies, patients received the following treatments.

In the asymptomatic group, patients without other pathologies were treated only with bLf capsules ranging from one (200 mg/day bLf) to five (1000 mg/day bLf) in number, according to the physician’s judgement. When the suggested dose was >200 mg/day, the capsules were divided into two or three daily administrations. BLf was usually taken before meals, in order to avoid protein degradation due to the low pH of gastric juice during digestion [47].

Asymptomatic patients with preexisting pathologies, in addition to bLf, received routine treatment for their specific diseases.

Among the symptomatic group, paucisymptomatic patients without preexisting pathologies and with symptoms such as fever and/or general discomfort and/or cough and/or myalgia and/or headache were treated with bLf ≥ 400 mg divided into two or three daily administrations associated with paracetamol 1000 mg 3 times/day until the fever subsided and/or ibuprofen 600 mg 2 times/day until the remission of symptoms and/or cortisone 25 mg/day until the remission of symptoms and/or azithromycin 500 mg/day for 6 days. In patients with preexisting pathologies, therapies for COVID-19, in addition to bLf administration of ≥400 mg divided into two or three daily administrations, were adapted according to personal pathology.

Moderate symptomatic patients without preexisting pathologies and with symptoms like cough and/or dyspnea without hypoxia and/or prolonged asthenia and/or decreased blood oxygen saturation, and eventually other symptoms were treated with bLf ≥ 400 mg divided into two or three daily administrations, associated with paracetamol 1000 mg 3 times/day until fever subsided and/or ibuprofen 600 mg 2 times/day until the remission of symptoms and/or cortisone 25 mg/day until the remission of symptoms and/or azithromycin 500 mg/day for 6 days.

In some moderate symptomatic patients with preexisting diseases, beyond the bLf treatment corresponding to ≥400 mg divided into two or three daily administrations and the already mentioned drugs (paracetamol, ibuprofen, cortisone, and azithromycin), 4000 IU/day of heparin was added until SARS-CoV-2 RNA negativization, depending on the cardiovascular risks.

Symptomatic patients with preexisting pathologies were obviously treated, in addition to bLf and the above-mentioned drugs, with the routine therapies specific for their diseases.

The two groups (bLf-treated versus untreated patients) had a ratio corresponding to 2:1, i.e., about two bLf-treated patients for every untreated patient. At the time of study design, this choice was dictated by the fact that an unequal patients distribution in favor of the active group could be useful to obtain more consistent safety information.

### 2.4. Evaluated Parameters

The main evaluated parameter was the time to achievement of SARS-CoV-2 RNA negativization. For this purpose, the patients were tested at 7 days after the first positive rRT-PCR nasopharyngeal swab. If they were still positive, the swab was repeated every week until SARS-CoV-2 RNA negativization. This procedure was performed for both groups, bLf-treated and untreated patients.

A second parameter evaluated was the median time to remission of symptoms. In particular, practitioners monitored home-based patients on a daily basis, asking them about their symptoms and general condition.

Hospitalization as well as safety and tolerability of bLf were daily monitored and evaluated.

### 2.5. Statistical Analysis

Data are summarized as mean and standard deviation (normally distributed data), median and interquartile range (IQR) (non-normally distributed data), or percent frequency (binary data), as appropriate. The comparison between two groups was performed by unpaired *t*-test (normally distributed data), Mann–Whitney U test non-normally distributed data, or Pearson’s chi-squared test (with continuity correction) (binary data), as appropriate. The association between two continuous variables was investigated by Spearman rank correlation coefficient (rho) and *p*-value.

The time to SARS-CoV-2 RNA negativization was investigated by multiple linear regression model as well as by Kaplan–Meier curves and multiple Cox regression analysis. The multiple linear regression model was applied to assess the extent of reduction in time to SARS-CoV-2 RNA negativization in bLf-treated patients versus those untreated, considering the effect of potential confounders. In this model, data were expressed as regression coefficients, standard errors of the regression coefficients, and *p*-value. The Kaplan–Meier analysis was used because it allows directly estimating the cumulative proportion of SARS-CoV-2 RNA negativization as a function of time. In this analysis, the curves (bLf-treated versus untreated patients) were compared by the Log Rank test. The Cox regression model was fitted to assess the hazard ratio of SARS-CoV-2 RNA negativization over time by adjusting for a series of potential confounders. In this analysis, data were expressed as hazard ratio, 95% confidence interval (CI), and *p*-value. In both multiple linear and Cox regression models, we considered the following as potential confounders: age, sex, comorbidity burden, and symptom severity. To account for the potential effect of treatment strategy changes over the course of the study on the effectiveness of bLf on the time to SARS-CoV-2 RNA negativization, a sensitivity analysis was performed by introducing the semester of enrolment in multivariate models. A *p* < 0.05 was considered statistically significant.

As for the days to symptom resolution, an effect modification analysis was undertaken to assess whether the effect of bLf treatment on this outcome variable is dependent on age. The effect modification by age of the effectiveness of bLf for reducing the days to symptom resolution (dependent variable) was investigated in a multiple linear regression model including the treatment with bLf (0 = no; 1 = yes), age, their interaction term (age × treatment) as well as sex, comorbidity burden, and symptom severity. The estimated difference (and 95% CI) of the days to SARS-CoV-2 RNA negativization between bLf-treated and untreated patients at predefined values of age was calculated by the linear combination method. This method specifically allows to estimate the between-groups differences in the days to symptom resolution by assuming that all patients of the study population had the same age values fixed at 40, 50, 60, 70, and 80 years. Data analysis was performed by SPSS for Windows IBM (version 22, Chicago, IL, USA).

## 3. Results

### 3.1. Baseline Demographic Characteristics for Patients 

**Figure 1:**
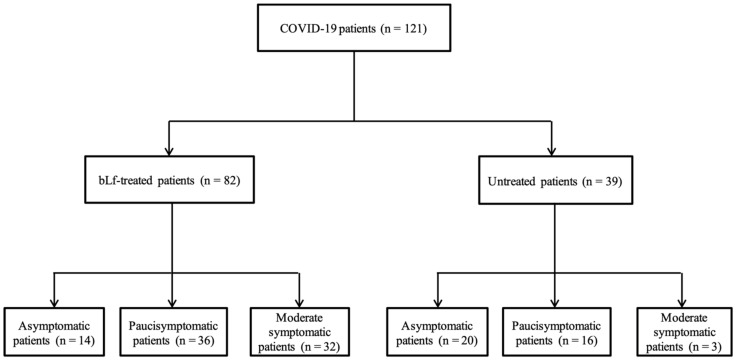
A total of 121 patients with confirmed COVID-19 infection at reverse transcriptase real-time (rRT)-PCR were involved in the study: 82/121 bovine lactoferrin (bLf)-treated and 39/121 bLf-untreated.

This study involved 121 patients as reported in the flowchart (Figure 1).

The patient’s characteristics are summarized in Table 1.

Eighty-two patients were treated with bLf, and the remaining 39 patients did not receive bLf treatment (Table 1). The two groups did not differ in age, gender, body weight, or comorbidity burden (Table 1 and Supplementary Materials Table S1). Conversely, the number of moderate symptomatic patients was significantly higher in bLf-treated patients than in those untreated (39.0% versus 7.7%) (Table 1). Among 82 bLf-treated patients, 14 asymptomatics were treated with bLf alone, while 36 paucisymptomatic and 32 moderate symptomatic patients were bLf-treated in association with paracetamol and/or ibuprofen and/or cortisone and/or azithromycin depending on their symptoms. With the exception of asymptomatics, in the control group, paucisymptomatics and moderate symptomatics received paracetamol and/or ibuprofen and/or cortisone and/or azithromycin depending on their symptoms.

**Figure 2:**
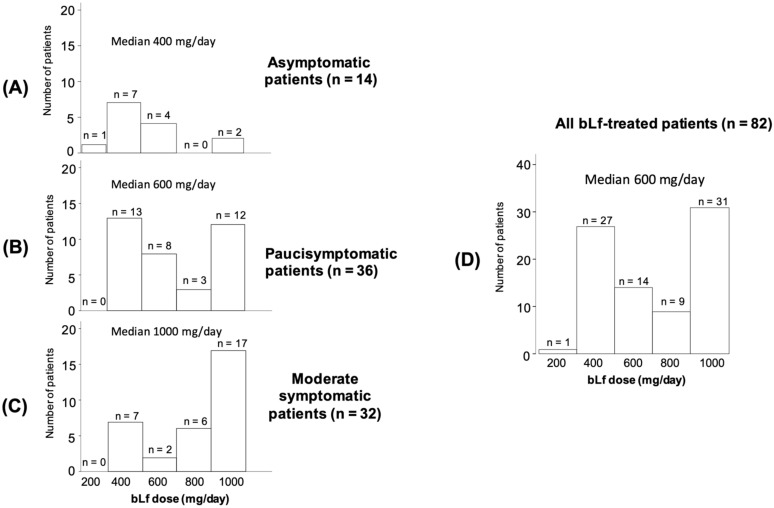
Bovine lactoferrin (bLf) doses in asymptomatic (**A**), paucisymptomatic (**B**), moderate symptomatic (**C**), and total patients (**D**).

Capsules, containing 200 mg of bLf each, were administered to bLf-treated patients. In asymptomatic patients, the median dose of bLf was 400 mg/day divided into two daily administrations (200 mg two times a day); in paucisymptomatics, the dose was 600 mg/day divided into 200 mg three times a day; and in moderate symptomatics, the dose was 1000 mg three times a day before meals (Figure 2A–C). In all bLf-treated patients, the median dose of bLf was 600 mg/day (IQR: 400–1000 mg/day) (Figure 2D). As expected, the median dose/day of bLf was significantly higher (*p* = 0.006) in moderate symptomatic patients (Figure 2C) than in those who were asymptomatic or paucisymptomatic (Figure 2A,B).

### 3.2. Time to SARS-CoV-2 RNA Negativization and Hospitalization Rates 

**Figure 3:**
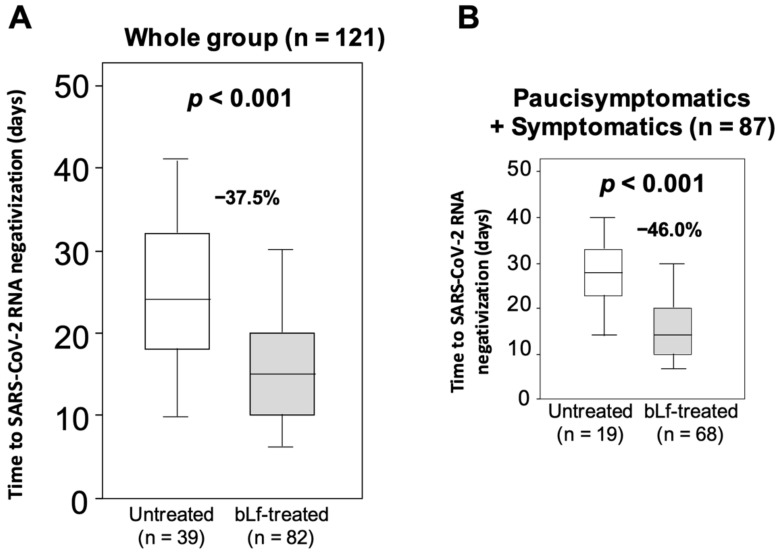
Box and Whisker plots of the days to SARS-CoV-2 RNA negativization in all bovine lactoferrin (bLf)-treated and untreated patients (**A**). The Box and Whisker plots are given in an analysis restricted to paucisymptomatic and moderate symptomatic patients (**B**). The ends of the box are the upper and lower quartiles. The median is indicated by the horizontal line inside the box. The 2.5th and the 97.5th percentile of the days to SARS-CoV-2 RNA negativization are also provided.

In all patients (*n* = 121), the median time to SARS-CoV-2 RNA negativization was 20 days (IQR: 12–25 days). The median value of days to SARS-CoV-2 RNA negativization was 37.5% lower (*p* < 0.001) in bLf-treated patients (median: 15 days, IQR: 10–20) than in those untreated (median: 24 days, IQR: 18–33) (Figure 3A). It is worth highlighting that the reduction in the days to SARS-CoV-2 RNA negativization in bLf-treated patients versus those untreated was of higher magnitude (−46.0%) in an analysis restricted to paucisymptomatic and moderate symptomatic patients (bLf-treated, median: 15 days, IQR: 10–20; untreated, median: 28 days, IQR: 23–33) (Figure 3B).

In asymptomatic patients (*n* = 34), no significant difference in the median time to SARS-CoV-2 RNA negativization was found (bLf-treated, median 15 days, IQR: 10–24; untreated, median: 19 days, IQR:14–30, *p* = 0.13), even though a reduced trend was observed in the bLf-treated patients (delta = 4 days).

**Figure 4:**
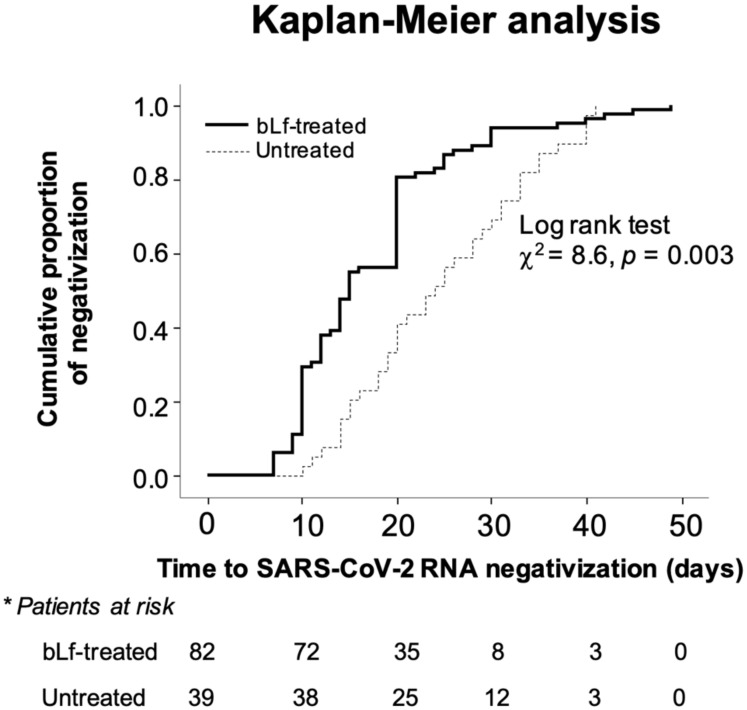
Cumulative proportion of SARS-CoV-2 RNA negativization as a function of time in patients treated and untreated with bovine lactoferrin (bLf). * Number of patients of both groups SARS-CoV-2 RNA-positive at different time points.

**Figure 5:**
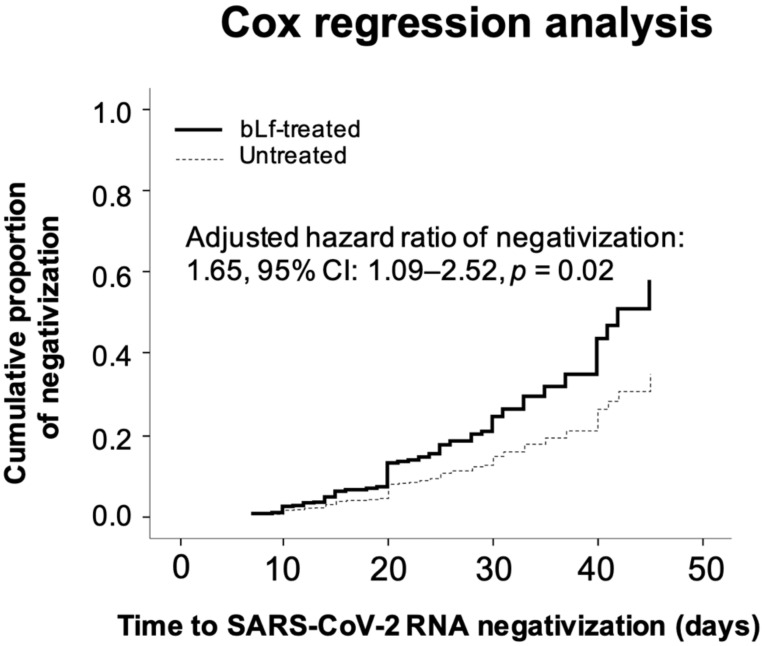
Cumulative proportion of SARS-CoV-2 RNA negativization as a function of time in patients treated and untreated with bovine lactoferrin (bLf), adjusted for confounders included in Table 2b. Data are presented as adjusted hazard ratio, 95% confidence interval (CI), and *p*-value.

The cumulative proportion of SARS-CoV-2 RNA negativization was significantly higher (*p =* 0.003) in bLf-treated patients than in those untreated (Figure 4). Moreover, the protective effect of bLf on time to SARS-CoV-2 RNA negativization (*p* < 0.001) was confirmed in a multiple linear regression model adjusting for age, gender, comorbidity burden, and severity of symptoms (Table 2a), as well as in a multiple Cox regression model (*p =* 0.02) adjusting for the same set of potential confounders (Table 2b and Figure 5). No dose-response effect or effect modification by age was found between the treatment with bLf and the time to SARS-CoV-2 RNA negativization.

Concerning the hospitalization rate, one patient was hospitalized in the untreated group and none in bLf-treated group. No adverse effects were observed in any of the bLf-treated patients across the entire study period.

### 3.3. Time to Symptom Resolution

**Figure 6:**
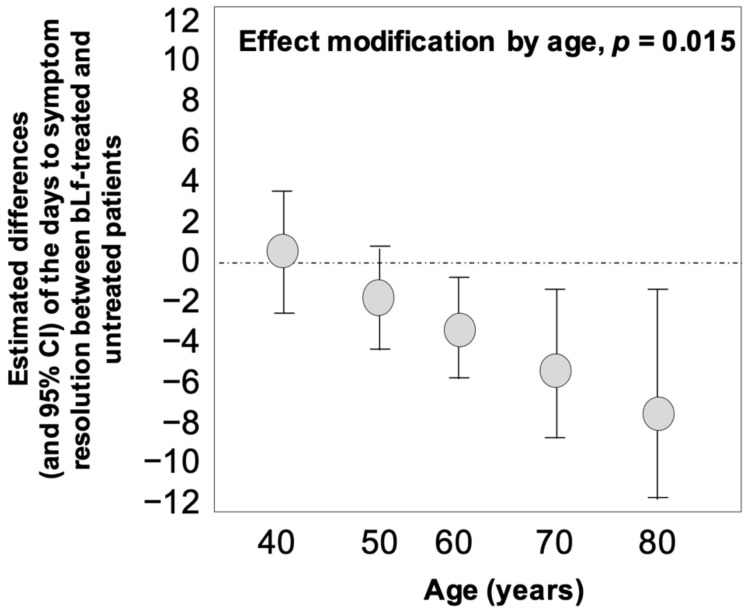
Effect modification by age on the effectiveness of bovine lactoferrin (bLf) in reducing the days to symptom resolution. Data are presented as estimated mean differences and 95% confidence interval (CI).

The time to symptom resolution did not significantly differ between bLf-treated (median 7 days, IQR: 5–8) and untreated patients (median 5 days, IQR: 4–10) (*p =* 0.50). However, starting from the patients aged 40, a significant correlation between age and effectiveness of bLf in reducing the days of symptoms (*p =* 0.015) was observed. The effectiveness of this treatment on symptom resolution was progressively higher in parallel with increasing age (Figure 6).

## 4. Discussion

In the absence of the availability of worldwide vaccination to fully counteract SARS-CoV-2 infection, factors related to innate immunity should be identified and assayed to improve natural host defenses. A prompt treatment could be the winning strategy to avoid or reduce as much as possible the real and serious problems related to this pandemic: patients’ hospitalization and the related mortality, especially concerning the elderly, obese, vaccinated individuals with only the first dose, and those suffering from chronic conditions.

Given the rapid transmission and severity of SARS-CoV-2 infections, in addition to the availability of vaccines, there is an urgent requirement for the discovery and development of supplementary antiviral agents.

In this regard, Lf, a natural glycoprotein belonging to the elements of innate immunity, could be a promising tool for the treatment of COVID-19 [18,32,33].

BLf, possessing about 70% sequence homology and identical functions with hLf [48], has been applied in in vitro and in vivo studies.

BLf possesses an antiviral activity [15,30,44] even against SARS-CoV-2 [22,33,34,45]. BLf, through iron-binding ability, inhibits both viral replication and formation of dangerous ROS. It is important to underline that bLf possesses a cationic feature, responsible for its binding to anionic compounds present on the surface of host cells and viruses, thus inhibiting viral adhesion and entry. A direct interaction between bLf and virus structural glycoproteins such as the SARS-CoV-2 spike [34,49], as well as between bLf and host receptors such as HSPGs [22], has been demonstrated. Finally, this glycoprotein is also able to enter inside host cells and to translocate into the nucleus, where it inhibits the transcription of proinflammatory cytokine genes [31]. In light of its ability to enter into the nucleus and decrease the synthesis of proinflammatory cytokines [29,31,32], bLf could strongly influence cytokine storm cascade activation in COVID-19 patients, avoiding systemic complications such as sepsis and coagulopathies and therefore decreasing disease severity. In particular, Lf is able to bind human plasminogen, thus regulating the coagulation cascade with the consequent anti-thrombotic activity [50], a very frequent complication of SARS-CoV-2 infection [28].

Collectively, this evidence has stimulated researchers to explore the role of bLf in different cell models, where its presence exerts protective and neutralizing activity against SARS-CoV-2, as well as partially inhibiting viral replication [22,34,45]. Following these in vitro results, for the first time, the efficacy of bLf in liposomal form against SARS-CoV-2 in COVID-19 patients has been demonstrated [33].

Here, we report a small retrospective study on the efficacy oral administration of bLf, unloaded in liposomes, on asymptomatic, paucisymptomatic and moderate symptomatic COVID-19 patients. The range in doses was chosen from data published on bLf efficacy against anemia of inflammation on hereditary thrombophilic pregnant women with high levels of serum IL-6 (100–200 mg two times a day) [40,51] as well as on COVID-19 patients (200 mg five times a day) [33]. Moreover, the bLf doses were different according to the severity of infection as well as the presence of comorbidities. The bLf median dosage was two capsules a day in asymptomatic patients, three capsules a day in paucisymptomatic patients, and five capsules a day in moderate symptomatic patients.

In all patients, the median number of days to SARS-CoV-2 RNA negativization was 37.5% lower in bLf-treated patients than in those untreated (15 versus 24 days). Remarkably, in an analysis restricted to paucisymptomatic and moderate symptomatic patients, the reduction in the number of days to SARS-CoV-2 RNA negativization was of a higher magnitude (−46.0%) in bLf-treated patients versus untreated ones (15 versus 28 days). Moreover, bLf oral administration unloaded in liposomes induces a time to SARS-CoV-2 RNA negativization similar to that observed with liposomal bLf (15 versus 14.25 days, respectively) [33]. As matter of fact, in gastric juice, bLf unloaded in liposomes and administered before meals undergoes a slight degradation [47]. Conversely, if bLf administration occurs after meals, a higher degradation was observed [47]. Of note, the degradation is in agreement with the loss of efficacy in counteracting iron and inflammatory homeostasis disorders [47]. Liposomal bLf in simulated gastric environment seems to maintain its integrity and biological efficacy, being resistant to enzymatic digestion by pepsin [52].

Overall, the time to symptom resolution did not significantly differ between bLf-treated and untreated patients. This finding most likely depends on the fact that in the bLf-treated group, there was a significantly higher number of moderate symptomatic patients than in the untreated group (39.0% versus 7.7%). Consequently, caution must be adopted while extending the study results to this group of patients. Furthermore, we observed a very interesting link between symptom reduction and age: there is a protective effect of bLf in reducing the time to symptom resolution with advancing age. This could be explained by the fact that the synthesis of hLf is under hormonal control [53], and therefore, it decreases with age. Moreover, another factor to be considered is that chronic low-grade inflammation is common in older individuals, and it is recognized as a strong risk factor for age-related disorders that cause high morbidity and mortality [54,55]. This state of chronic inflammation that correlates with aging, sometimes referred to as “inflamm-aging”, is a high-risk factor for the occurrence, progression, and complication of many diseases. Clinically, inflamm-aging is characterized by increased blood levels of several inflammatory biomarkers, including C-reactive protein, IL-6, IL-18, and tumor necrosis factor-α [56]. Lf shows a potent anti-inflammatory action, lowering the levels of some proinflammatory cytokines such as IL-6 which, at high levels, leads to iron homeostasis disorders and tissue injuries [31]. IL-6 blockage may contribute to counteracting severe and critical outcome in COVID-19 patients.

Based on preliminary data of our small retrospective study, bLf could be considered a putative supplementary treatment in asymptomatic, paucisymptomatic, and moderate symptomatic patients. It is important to underline that in our small COVID-19 population, no patients experienced a fatal outcome. In our opinion, this is an encouraging preliminary result to be confirmed in a large number of COVID-19 patients. Even if the number of treated patients was small, they were characterized by a high proportion of subjects aged > 50 years; the use of bLf seems to improve outcome in patients affected by COVID-19, including those with more symptoms, comorbid diseases, and advanced age. Moreover, one patient was hospitalized in the untreated group and none in the bLf-treated group. No adverse effects in any bLf-treated patients were observed across the study period. On the other hand, our findings are in agreement with those reported in more than one thousand pregnant women treated with bLf [31,57].

Concerning the mechanism(s) of action of the antiviral activity of bLf, this glycoprotein acts through direct interaction with SARS-CoV-2 spike [34,49]; interaction with host cells HSPGs [22]; and anti-inflammatory activity restoring the iron homeostasis disorders [33,45], which, if not restored, leads to intracellular iron overload, favoring viral replication and infection [58].

This real-life study, representing the population normally treated and monitored daily in clinical practice, does present several limitations that need to be mentioned.

First, the isolated SARS-CoV-2 strains were not sequenced, and the number of bLf-treated patients was low. Second, no randomization was performed. These limitations could be overcome, because the increase in the vaccinated population related to a lower number of COVID-19 positive patients could lead to a higher degree of SARS-CoV-2 strain sequencing. These data could add pivotal information on the ability of bLf to also counteract infections by SARS-CoV-2 variants.

Obviously, a larger number of bLf-treated versus untreated patients with a more balanced distribution of males and females and reporting information on baseline viral load are required to confirm these preliminary observations.

Even if real-life studies are more frequently undertaken in recent years, randomized controlled trials (RCTs) represent the most effective valuation of a therapeutic intervention.

As a matter of fact, the observational retrospective nature of our study precludes the possibility of drawing definitive conclusions about the efficacy of bLf in COVID-19 patients. For this reason, further RCTs on wider number of COVID-19 patients are required to confirm our preliminary observations on the efficacy of bLf treatment.

## 5. Conclusions

To our knowledge, this is the first preliminary retrospective study including SARS-CoV-2 patients treated with unloaded liposome bLf, and our observations will be useful for further, wider future studies. In conclusion, the results obtained highlight a lower number of days to SARS-CoV-2 RNA negativization in bLf-treated patients as well as a link among bLf treatment, reduction in symptoms, and age, which represent undoubtedly the basis for enriching the limited literature on bLf effectiveness for COVID-19 treatment.

## Figures and Tables

**Table 1:** Main characteristics of patients.

Variables	Total Population (*n* = 121)	Patients Treated with bLf (*n* = 82)	Patients Untreated with bLf (*n* = 39)	** *p*-Value
Age (years)	46.6 ± 18.6	47.7 ± 19.7	44.5 ± 16.2	0.36
Male gender *n*. (%)	47 (39.0%)	32 (39.0%)	15 (38.5%)	0.95
Body weight (kg)	66.6 ± 12.6	65.7 ± 12.2	68.4 ± 13.2	0.29
* Comorbidities *n*. (%) 0 1 2 ≥3	86 (71.1%) 21 (17.4%) 9 (7.4%) 5 (4.1%)	57 (69.5%) 17 (20.7%) 5 (6.1%) 3 (3.7%)	29 (74.4%) 4 (10.3%) 4 (10.3%) 2 (5.1%)	0.47
Asymptomatic Paucisymptomatic Moderate symptomatic	34 (28.1%) 52 (43.0%) 35 (28.9%)	14 (17.1%) 36 (43.9%) 32 (39.0%)	20 (51.3%) 16 (41.0%) 3 (7.7%)	<0.001

* Comorbidities include: overweight/obesity, human immunodeficiency virus (HIV), arthritis, rheumatic disease, asthma, chronic obstructive pulmonary disease, chronic bronchitis, hypertension, diabetes, cardiomyopathy, atrial fibrillation, cardiovascular disease. ** The comparison between the two groups (bLf-treated versus untreated patients) was performed by unpaired *t*-test (for age and body weight) and Pearson’s chi-squared test (for gender, comorbidities, and symptoms). Age and body weight were summarized as mean and standard deviation. bLf = bovine lactoferrin.

**Table 2:** Multiple linear regression (a) and multiple Cox regression (b) models of time to SARS-CoV-2 RNA negativization.

(a) Multiple linear regression model		
**Variables**	**Regression Coefficients ± SE**	***p*-Value**
Treatment with bLf (0 = no; 1 = yes)	−8.00 ± 1.90	<0.001
Age (years)	0.09 ± 0.05	0.07
Male gender	0.41 ± 1.72	0.81
* Comorbidity burden	−0.66 ± 1.10	0.55
** Severity of symptoms	0.72 ± 1.23	0.56
(b) Multiple Cox regression model		
**Variables**	**Hazard Ratio and 95% CI**	***p*-Value**
Treatment with bLf (0 = no; 1 = yes)	1.65 (1.09–2.52)	0.02
Age (years)	0.99 (0.98–1.01)	0.08
Male gender	0.95 (0.65–1.39)	0.78
* Comorbidity burden	1.12 (0.86–1.45)	0.39
** Severity of symptoms	0.97 (0.74–1.28)	0.84

* Codified as 0, 1, 2, and ≥3. ** Codified as 0 (asymptomatic), 1 (paucisymptomatic), and 2 (moderate symptomatic). Forcing the semester of treatment into the two models did not affect the strength of the bLf effect on the time to SARS-CoV-2 RNA negativization in either the linear regression model (regression coefficient: −8.2 ± 2.0, *p* < 0.001) or the Cox regression analysis (hazard ratio: 1.91, 95% CI: 1.19–3.06, *p =* 0.007). bLf = bovine lactoferrin; CI = confidence interval; SE = standard error.

## Data Availability

The data presented in this study are available in the article and Supplementary Material.

## Supplementary Materials

The following are available online at https://www.mdpi.com/article/10.3390/jcm10184276/s1, Table S1. Burden of comorbidities by treatment groups.
