# Supplementary material for: Ambulatory COVID-19 Patients Treated with Lactoferrin as a Supplementary Antiviral Agent: A Preliminary Study
Source: J Clin Med. 2021 Sep 21;10(18):4276. doi: 10.3390/jcm10184276 (PMC8469309; doi:10.3390/jcm10184276)
Supplement: Supplementary file 1 [file jcm-10-04276-s001.zip › jcm-1336466-supplementary.pdf]

Supplementary Materials

Table S1. Burden of comorbidities by treatment groups.

Type of Comorbidity	Patients Treated with bLf (*n* = 82)	Patients Untreated with bLf (*n* = 39)	*p*-Value
Overweight/obesity, *n*.	7	3	1.00
Human immunodeficiency virus (HIV), *n*.	0	2	0.10
Arthritis/ rheumatic disease, *n*.	2	0	1.00
Asthma, *n*.	3	2	0.66
BPCO/Chronic bronchitis, *n*.	2	0	1.00
Hypertension, *n*.	11	6	0.77
Diabetes, *n*.	4	3	0.68
Cardiomyopathy, *n*.	3	0	0.55
Atrial fibrillation, *n*.	1	0	1.00
Cardiovascular disease, *n*.	3	2	0.66

BPCO = Chronic obstructive pulmonary disease; bLf = bovine lactoferrin.
